# Impact of fee subsidy policy on perinatal health in a low-resource setting: A quasi-experimental study

**DOI:** 10.1371/journal.pone.0206978

**Published:** 2018-11-08

**Authors:** Ivlabèhiré Bertrand Meda, Alexandre Dumont, Seni Kouanda, Valéry Ridde

**Affiliations:** 1 Département Biomédical et Santé Publique, Institut de Recherche en Sciences de la Santé (IRSS/CNRST), Ouagadougou, Burkina Faso; 2 École de Santé Publique de l’Université de Montréal (ESPUM), Montréal, Canada; 3 Institut de Recherche en Santé Publique de l’Université de Montréal (IRSPUM), Montréal, Canada; 4 Institut Africain de Santé Publique (IASP), Ouagadougou, Burkina Faso; 5 IRD (French Institute For Research on sustainable Development), CEPED (IRD-Université Paris Descartes), Universités Paris Sorbonne Cités, ERL INSERM SAGESUD, Paris, France; UMC Utrecht Julius Centrum, NETHERLANDS

## Abstract

**Background:**

A national subsidy policy was introduced in 2007 in Burkina Faso to improve financial accessibility to facility-based delivery. In this article, we estimated the effects of reducing user fees on institutional delivery and neonatal mortality, immediately and three years after the introduction of the policy.

**Methods:**

The study was based on a quasi-experimental design. We used data obtained from the 2010 Demographic and Health Survey, including survival information for 32,102 live-born infants born to 12,474 women. We used a multilevel Poisson regression model with robust variances to control for secular trends in outcomes between the period before the introduction of the policy (1 January, 2007) and the period after. In sensitivity analyses, we used two different models according to the different definitions of the period “before” and the period “after”.

**Results:**

Immediately following its introduction, the subsidy policy was associated with increases in institutional deliveries by 4% (RR = 1.04, 95% CI: 0.98–1.10) in urban areas and by 12% (RR = 1.12, 95% CI: 1.04–1.20) in rural areas. The results showed similar patterns in sensitivity analyses. This effect was particularly marked among rural clusters with low institutional delivery rates at baseline (RR = 1.44, 95% CI: 1.33–1.55). It was persistent for 42 months after the introduction of the policy but these increases were not statistically significant. At 42 months, the delivery rates had increased by 26% in rural areas (RR = 1.26; 95% CI: 0.86–1.86) and 13% (RR = 1.13; 95% CI: 0.88–1.46) in urban areas. There was no evidence of a significant decrease in neonatal mortality rates.

**Conclusion:**

The delivery subsidy implemented in Burkina Faso is associated with short-term increases in health facility deliveries. This policy has been particularly beneficial for rural households.

## Introduction

In many developing countries, childbirth in health facilities is considered to be an assisted delivery performed by a qualified health personnel [1]. Hence, skilled attendance at delivery is considered as one of the most effective interventions to fight against maternal and neonatal mortality, as it allows an early detection of the deadliest complications [2]. Unfortunately, in Sub-Saharan Africa, only 46% of childbirths occurred in a health facility in 2015 [3].

Since the early 2000s, several low-income countries have introduced public policies to reduce the cost barrier to access to maternal and infant health care [4].

Considerable literature supports the association of these policies with an increase in facility-based deliveries [5–13]. This increase in institutional deliveries varies depending on the area of residence [8] (rural *versus* urban), the mother’s education level [8, 9] and the household wealth [7, 8].

Systematic reviews [14–16] have underlined the methodological weaknesses of most of these studies (i.e., weak designs, inappropriate analytical methods, and short observation time periods). Additionally, few studies have focused on the effects of these policies on equitable access to care [5–9, 17] and the long-term effects; only three studies have assessed the association with neonatal mortality at the population level [10, 18, 19].

Although these policies are recognized to be associated with an increase in the use of health services, quantification and persistence over time have not been well defined. Studies using the same population data have shown different results for the same country (e.g., in Burkina Faso and Ghana in 2016) [6, 8, 10]. Moreover, national level aggregated analyses [6, 8] do not include the heterogeneity of a country’s effects.

The purpose of this study is to assess the changes in institutional delivery and neonatal mortality rates following the introduction of a national subsidy for deliveries and emergency obstetric and neonatal care in Burkina Faso. Additionally, this study investigates the persistence of these changes three years after policy implementation and their heterogeneity based on the area of residence, household income level and the woman’s education level.

## Materials and methods

### Design, setting and study population

Burkina Faso is a West African low-income country with a population of more than 19 million inhabitants in 2017. The institutional delivery rate increased from 38% in 2003 to 66% in 2010, and the neonatal mortality rate ranged from 31 per 1,000 in 2003 to 28 per 1,000 live births in 2010 [20].

We used data from the latest Demographic and Health Survey (DHS) conducted in Burkina Faso in 2010. The DHSs are regular cross-sectional household survey conducted in several low-income countries. The DHS methodology, questionnaires and reports are available online (http://dhsprogram.com/).

The women’s questionnaire helps collect sociodemographic characteristics and the reproductive life history of all 15- to 49-year-old women from the surveyed households. The vital status of each live-born child prior to the survey is collected for each woman. The age at death (days, months or years) is recorded when a child dies.

Using a quasi-experimental approach, we tested the impact of the delivery policy subsidy by comparing outcomes between the period before and the period after the introduction of the policy. The subsidy was implemented at the national level; thus, formation of a control group of women from subsidy-free areas was impossible.

Of the 14,947 households selected, 96.5% (14,424) were surveyed. Of the 17,363 women aged 15 to 49 identified in these surveyed households, 17,087 responded to the women’s questionnaire, yielding a response rate of 98.4%. Most of the information is self-reported. The data are organized depending on the following ascending hierarchical structure: new-born, woman, household, household cluster and region.

### Study variables

#### Exposure variable: The national subsidy policy

The national subsidy for deliveries and emergency obstetric and neonatal care represented the exposure and has been described elsewhere [12, 21]. Briefly, this national subsidy policy covered approximately 60 to 80% of direct medical expenses (depending on whether the delivery occurred at a hospital or a health centre). The remaining 20 to 40% of the expenses were borne by the parturient. This policy was adopted in October 2006 and was officially implemented on 1 January, 2007 [12]. However, depending on the health district, the policy was introduced between January and April 2007 [7, 12, 22]. Several studies [12, 23, 24] indicated that this national subsidy policy was not evenly established throughout the country. The DHS data do not allow a connection between household clusters and health districts. The subsidy exposure was set to 1 for exposed and 0 for non-exposed, indicating whether each live birth occurred after the introduction of the subsidy policy.

#### Outcomes

Two binary variables were considered for each live birth included in the study (birth place and vital status at 28 days of life).

The place of birth was given a value of 1 for a health facility delivery (public or private) and 0 for delivery outside a health facility. The information is available for live births that occurred during the 5 years prior the survey. Multiple births were considered as one delivery.

The new-born’s vital status was coded as 1 for new-borns who died within the first 28 days of life and 0 for new-borns who remained alive [25]. The information for age at death is available for 98.2% of children who died; the remaining 1.8% was imputed. Three causes (infections, complications of preterm birth and intrapartum-related neonatal death) account for 80% of neonatal deaths in low-income countries, and access to assisted delivery can significantly reduce mortality related to these causes [25]. Therefore, the evaluation of this outcome does not require a latency period since a significant increase in health facility deliveries can result in an immediate decrease in neonatal mortality if obstetric and neonatal care are good quality.

Only live births that occurred during the 10 years prior to the survey were included in the neonatal mortality analysis to consider the following: first, the cross-sectional nature of certain control variables, and second, whether this effect contributed to limiting a possible secular trend in variation that could be overshadowed by a long pre-intervention period.

#### Interaction and control variables

Based on the literature, [6–9, 19] the following four effect-modifier variables were considered: the parturient’s education and literacy, the area of residence (rural vs urban) and the household wealth. The woman’s education comprised three categories [7–9] (no education, primary and secondary or higher). Literate women (those who could partially or fully read a sentence) were differentiated from illiterate women. We assume that the categorization of this second variable should be more homogeneous, since women with similar education levels may have different reading skills. Household wealth is a wealth index ranking of all surveyed households by income quintiles. This index was provided by DHS and was built by principal components analysis [26] using the source of drinking water, type of sanitary system, housing characteristics and the household’s possession of some durable goods (i.e., television, telephone, and refrigerator).

Several variables associated with health facility delivery and/or neonatal death reported in the literature [1, 27] were considered as control variables. These variables are the maternal age at the time of delivery (categorized by 5-year intervals), number of births per delivery (single vs multiple), birth order (first, 2 to 4th and 5th or more), interval since precedent birth (36 months or more, less than 36 months and first delivery), new-born gender (female vs male) and the woman’s occupation status during the survey period (working vs not working). The work category included paid or unpaid work. The woman’s marital status (98.5% married) was discarded because it was non-selective.

Breastfeeding and birth weight variables were not included because they were only reported for births in the 5 years preceding the survey. In addition, birth weight accounted for more than one-third (34.6%) of the missing data. The distance of households to the nearest health centre was not reported.

### Data analysis

The unit of analysis is the new-born. A modified multilevel Poisson regression [28, 29] analysis was performed to obtain the rates directly. Three levels were considered: level 1 (new-born), level 2 (woman or household) and level 3 (household cluster). A segmented regression analysis including a continuous time variable (in months) counted from the start to the end of the observation period was performed to isolate the secular trend and make adjustments for autocorrelation of the observations [30, 31]. A variable (post-time) with value 0 before the subsidy and counting the number of months since the introduction of the subsidy was also included to assess the change in the slope induced by the subsidy [30, 31]. In addition to the random intercepts, variable coefficients allocated to the subsidy, time and post-time variables were considered depending on the clusters [32]. The choice of a model with random intercept and coefficients is also based on the results of a previous study that showed that the effects of this policy varied by district [33]. Education and literacy were separately introduced into the models due to their strong correlation. Based on these specifications, the following 3-level models were analysed:

$$\begin{array}{l} \text{log}\left(Y_{ijk}\right)=\left(\beta_{0}+\gamma_{0jk}+\gamma_{00k}\right)+\left(\beta_{1}+\gamma_{1k}\right)T_{ijk}+\left(\beta_{2}+\gamma_{2k}\right)I_{ijk}+\left(\beta_{3}+\gamma_{3k}\right)posttime_{ijk}\\ +\beta_{4}rural_{k}+\beta_{5}SES_{jk}+\beta_{6}Educ_{jk}\\ +\beta_{7}I_{ijk}*rural_{k}+\beta_{8}I_{ijk}*SES_{jk}+\beta_{9}I_{ijk}*Educ_{jk}+\beta_{10}X_{ijk} \end{array}$$

where i = i^th^ new-born, j = j^th^ woman or household, k = k^th^ household cluster, Y_ijk_ = outcome status, T_ijk_ = length of time since the beginning of the observation (January 2000 or June 2005 depending on the outcome), post-time_ijk_ = length of time since the introduction of the subsidy, I_ijk_ = subsidy status, Rural_k_ = cluster localization, SES_jk_ = household wealth, Educ_jk_ = education or woman’s literacy, and X_ijk_ = control variables.

For this model, *β*_1_ estimates the secular trend, *β*_2_ represents the immediate effect of the subsidy, and *β*_3_ evaluates the change in the secular trend after the introduction of the subsidy. The sum of *β*_1_ and *β*_3_ represents the secular trend after the introduction of the subsidy. *γ*_0*jk*_ and *γ*_00*k*_ represent the level 2 and 3 random intercepts, and *γ*_1*k*_, *γ*_2*k*_ and *γ*_3*k*_ constitute the level 3 random effects related to *β*_1_, *β*_2_ and *β*_3_, respectively. The interactions between the subsidy and the area of residence, the household wealth and the woman’s education are respectively estimated by coefficients *β*_7_, *β*_8_ and *β*_9_.

By considering 1 January 2007 as the subsidy start date, some non-exposed births will be classified as exposed. To assess the consequences of these misclassifications on the results, we conducted two sensitivity analyses: the first one considered 1 April 2007 as the subsidy introduction date. This strategy leads to the classification of certain exposed births as non-exposed births. The second one excluded births that occurred between 1 January and 31 March 2007, as suggested by some authors [34, 35], because whether these births were exposed or not could not be determined.

The three-level models were tested against two-level models with the cluster at level 2. In the birth place analysis, the woman and household levels showed no variance, and the analysis was therefore limited to two levels. The interactions were evaluated after controlling for confounding factors. The three interactions were tested together and then separately for each dependent variable. The interaction between the area of residence and the subsidy for the birth place was the only significant interaction, and the analyses were conducted separately in subgroups for urban births and rural births. Over-dispersion was tested by introducing a random intercept at level 1 (new-born) [36]. Standard errors were adjusted for regional level clustering for all analyses.

From the final models, a seasonal variable representing the calendar month of birth was introduced to control for potential seasonal fluctuations [31]. Only the tests of interactions, random effects and hierarchical structure used the likelihood ratio test with a p-value at the 0.05 threshold. The other comparisons (confounding and seasonality) focused on the subsidy-modifying coefficient of at least 10%. We checked the multicollinearity of independent variables by using the variance inflation factors. The likelihood ratio test was used to test the fit of nested models, and we analysed the residual statistics to assess the fit of the models.

A model-based standardization [37, 38] was conducted to calculate the rate ratios and rate differences associated with the subsidy for different periods (0 to 42 months) at the population level. Each new-born’s probability of death or birth in a health centre was predicted for each period under two scenarios (with and without the subsidy) and by replacement of the time and post-time variables with their corresponding time values. These predictions incorporated the random-effects predicted values (obtained by empirical Bayes prediction) [36] for each woman, household and cluster [37]. Then, the probabilities were averaged for each scenario and period, and their ratios and differences were calculated. Calculations were also performed for the three and two-level models, and similar results were obtained. The results are reported here for the two-level models for parsimony. Confidence intervals were obtained using the delta method [38, 39].

The analyses were conducted with the Generalized Linear Latent and Mixed Models (GLLAMM) [36] program in Stata SE 14.2 (StataCorp LP).

## Results

**Fig 1:**
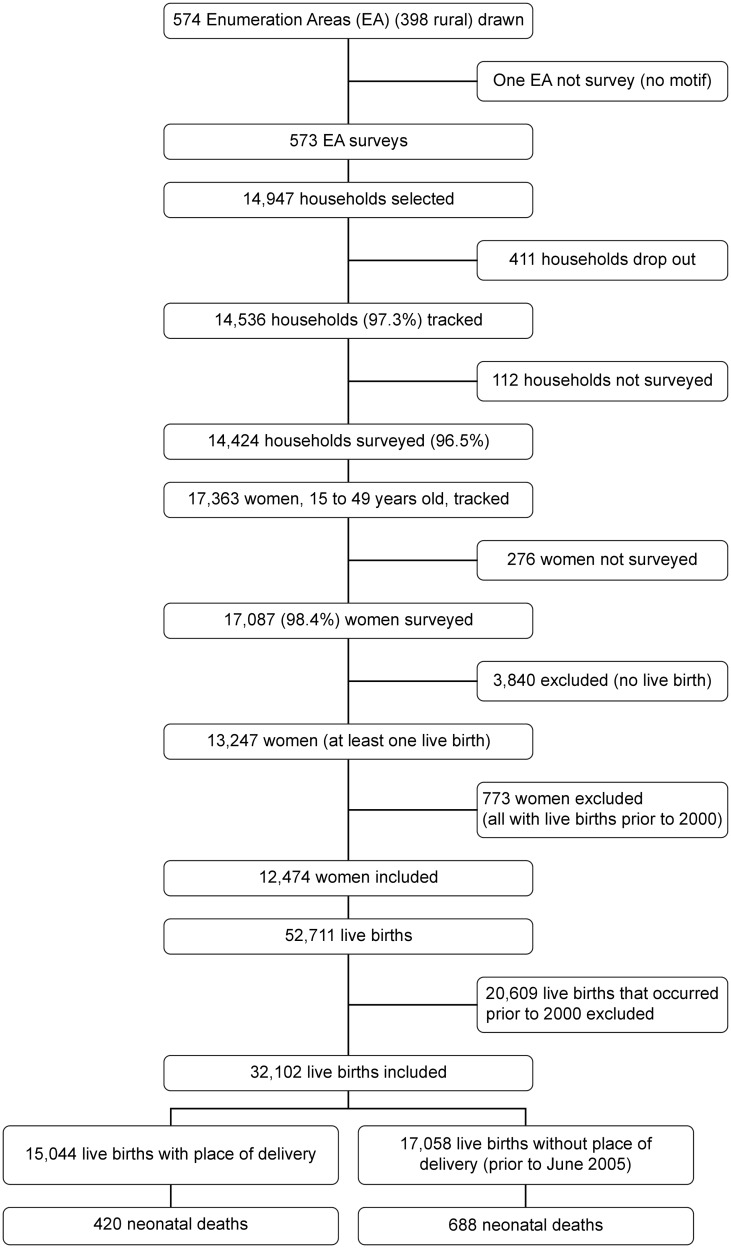
Flow diagram of the sample selection process used in the analysis.

Fig 1 shows a flow diagram of the selection process used for the sample included in the analysis. The analysis involved 32,102 live births registered between January 2000 and December 2010. Only the 15,044 births within the 5-year period before the survey provided birthplace details. Therefore, the analysis of the delivery rates in health facilities was performed on 14,753 deliveries since multiple births were considered as a single [10]. The 12,474 women were from 9,996 households. Among these women, 6,967 had live births in both the pre- and post-intervention periods. The number of births per woman ranged from 1 to 8 with a median of 3, and the number of women per household ranged from 1 to 7 with a median of 1.

The characteristics of the new-borns, women and households are presented in Table 1 for births occurring from January 2000 to December 2010 (see S1 Table for the births occurring from June 2005 to December 2010). The sociodemographic characteristics of the samples were broadly similar in the pre-and post-intervention periods.

**Table 1:** Sociodemographic and economic characteristics of live births that occurred in Burkina Faso between January 2000 and December 2010 (Demographic and Health Survey 2010).

Characteristic	Pre-subsidy introduction* n = 20,513	Post-subsidy introduction* n = 11,589	Total n = 32,102
Neonatal deaths	803 (3.9)	305 (2.6)	1,108 (3.5)
New-born’s sex (male)	10,551 (51.2)	5,924 (51.1)	16,435 (51.2)
Number of births (multiple)	673 (3.3)	461 (4.0)	1,134 (3.5)
Woman’s age (years)			
• 15–19	2,860 (13.9)	1,329 (11.5)	4,189 (13.0)
• 20–24	5,915 (28.8)	3,096 (26.7)	9,011 (28.1)
• 25–29	4,833 (23.6)	3,014 (26.0)	7,847 (24.4)
• 30–34	3,731 (18.2)	2,101 (18.1)	5,832 (18.2)
• 35–39	2,418 (11.8)	1,365 (11.8)	3,783 (11.8)
• 40–49**	756 (3.7)	684 (5.9)	1,440 (4.5)
Previous birth interval			
• 36 months or higher	6,392 (31.2)	4,975 (42.9)	11,367 (35.4)
• Less than 36 months	10,082 (49.1)	4,458 (38.5)	14,540 (45.3)
• First birth	4,039 (19.7)	2,156 (18.6)	6,195 (19.3)
Woman’s literacy (illiterate)	18,241 (88.9)	9,972 (86.0)	28,213 (87.9)
Woman’s education			
• None	17,854 (87.0)	9,641 (83.2)	27,495 (85.7)
• Primary	1,876 (9.2)	1,312 (11.3)	3,188 (9.9)
• Secondary or higher	783 (3.8)	636 (5.5)	1,419 (4.4)
Woman’s occupation (worked)	16,811 (82.0)	9,173 (79.2)	25,984 (80.9)
Household wealth			
• Poorest	4,375 (21.3)	2,250 (19.4)	6,625 (20.6)
• Poorer	4,377 (21.4)	2,456 (21.2)	6,833 (21.3)
• Middle	4,374 (21.3)	2,578 (22.2)	6,952 (21.7)
• Richer	4,368 (21.3)	2,500 (21.6)	6,868 (21.4)
• Richest	3,019 (14.7)	1,805 (15.6)	4,824 (15.0)
Place of residence (rural)	16,337 (79.6)	9,119 (78.7)	25,456 (79.3)

* Subsidy start date: 1 January 2007

**The 40–44 and 45–49 age groups were merged due to their low numbers.

Tables 2 and 3 present the assessments of the effect of the subsidy on institutional delivery by residence. No variance was found between the clusters in the urban stratum, and a simple Poisson regression was performed. According to primary and sensitivity analyses, the coefficient estimating the subsidy immediate effect varied between 0.0973 (p<0.001) and 0.1716 (p<0.001) in rural areas and between 0.0386 (p = 0.175) and 0.0503 (p = 0.035) in urban areas. The change in the secular trend after the introduction of the subsidy was not significant irrespective of the urban or rural setting and the scenario considered.

**Table 2:** Estimates for different two-level Random-coefficient (RC) Poisson regression models for the effects of the national subsidy on birth attendance in a health facility in rural Burkina Faso (Demographic and Health Survey 2010).

Characteristics	Primary analysis*			Sensitivity analysis 1**			Sensitivity analysis 2***		
	Estimate	SE	P-value	Estimate	SE	P-value	Estimate	SE	p-value
Non-adjusted coefficient									
Subsidy	0.2606	0.0528	<0.001	0.2510	0.0501	<0.001	0.2684	0.0540	<0.001
Fully adjusted coefficients									
Intercept	-0.9234	0.1766	< 0.001	-0.9683	0.1809	<0.001	-0.9211	0.1778	<0.001
Subsidy	0.1388	0.0385	< 0.001	0.0973	0.0293	<0.001	0.1716	0.0390	<0.001
Time	0.0050	0.0042	0.238	0.0097	0.0031	0.002	0.0046	0.0042	0.266
Post-time	0.0027	0.0040	0.495	-0.0022	0.0029	0.444	0.0027	0.0040	0.495
Illiterate (ref = Literate)	-0.0999	0.0209	<0.001	-0.1005	0.0207	<0.001	-0.1068	0.0188	<0.001
Household wealth (ref = Poorest)									
• Poorer	0.1069	0.0254	<0.001	0.1065	0.0255	<0.001	0.1194	0.0250	<0.001
• Middle	0.1728	0.0385	<0.001	0.1719	0.0386	<0.001	0.1812	0.0407	<0.001
• Richer	0.2112	0.0436	<0.001	0.2101	0.0435	<0.001	0.2168	0.0442	<0.001
• Richest	0.2752	0.0517	<0.001	0.2742	0.0519	<0.001	0.2828	0.0518	<0.001
Multiple births (ref = Single)	0.1136	0.0468	0.015	0.1140	0.0474	0.016	0.0995	0.0487	0.041
Woman’s age (ref = 15–19)									
• 20–24	-0.0455	0.0234	0.052	-0.0450	0.0237	0.058	-0.0434	0.0262	0.098
• 25–29	-0.0662	0.0336	0.049	-0.0663	0.0339	0.050	-0.0664	0.0346	0.055
• 30–34	-0.0358	0.0338	0.288	-0.0358	0.0337	0.287	-0.0468	0.0379	0.217
• 35–39	-0.0223	0.0467	0.634	-0.0234	0.0467	0.616	-0.0270	0.0498	0.588
• 40–49	-0.0267	0.0473	0.572	-0.0275	0.0473	0.561	-0.0220	0.0462	0.635
Birth order (ref = first)									
• 2^nd^ to 4th	-0.0811	0.0198	<0.001	-0.0817	0.0199	<0.001	-0.0791	0.0197	<0.001
• 5th or higher	-0.1244	0.0354	<0.001	-0.1241	0.0355	<0.001	-0.1205	0.0357	<0.001
Worked (ref = Not worked)	0.0948	0.0597	0.112	0.0948	0.0602	0.115	0.1006	0.0622	0.106
Random parts									
$\sqrt{\psi_{11}}$ (Intercept)	0.7682			0.7573			0.7518		
$\sqrt{\psi_{22}}$ (Subsidy)	0.0819			0.0755			0.1015		
$\sqrt{\psi_{33}}$ (Time)	0.0044			0.0138			0.0041		

*Primary analysis: Introduction of the policy estimated at 1 January 2007.

**Sensitivity analysis 1: Introduction of the policy estimated at 1 April 2007.

***Sensitivity analysis 2: Births between 1 January and 31 March 2007 were deleted (Subsidy introduction, 1 January 2007).

**Table 3:** Estimates for different Poisson regression models for the effects of the national subsidy on birth attendance in a health facility in urban Burkina Faso (Demographic and Health Survey 2010).

Characteristics	Primary analysis*			Sensitivity analysis 1**			Sensitivity analysis 2***		
	Estimate	SE	P-value	Estimate	SE	P-value	Estimate	SE	p-value
Non-adjusted coefficient									
Subsidy	0.0688	0.0234	0.003	0.0735	0.0220	0.001	0.0734	0.0239	0.002
Fully adjusted coefficients									
Intercept	-0.5405	0.1202	<0.001	-0.5467	0.1256	<0.001	-0.5269	0.1127	<0.001
Subsidy	0.0386	0.0285	0.175	0.0417	0.0177	0.018	0.0503	0.0239	0.035
Time	-0.0004	0.0024	0.860	0.004	0.0018	0.839	-0.0004	0.0024	0.857
Post-time	0.002	0.0026	0.445	0.0010	0.0020	0.622	0.0018	0.0028	0.521
Illiterate (ref = Literate)	-0.0604	0.0180	<0.001	-0.0605	0.0180	<0.001	-0.0581	0.0179	0.001
Household wealth (ref = Poorest)									
• Poorer	0.2458	0.0556	<0.001	0.2465	0.0555	<0.001	0.2313	0.0536	<0.001
• Middle	0.3017	0.0865	<0.001	0.3015	0.0861	<0.001	0.2888	0.0867	<0.001
• Richer	0.3931	0.0845	<0.001	0.3932	0.0841	<0.001	0.3797	0.0758	<0.001
• Richest	0.4239	0.0969	<0.001	0.4232	0.0964	<0.001	0.4095	0.0883	<0.001
Multiple births (ref = Single)	0.0113	0.0537	0.834	0.0094	0.0537	0.861	0.0078	0.0542	0.885
Woman’s age (ref = 15–19)									
• 20–24	0.0451	0.0271	0.096	0.0443	0.0266	0.096	0.0425	0.0273	0.120
• 25–29	0.0580	0.0260	0.026	0.0575	0.0256	0.025	0.0520	0.0257	0.043
• 30–34	0.0582	0.0333	0.080	0.0578	0.0327	0.077	0.0556	0.0310	0.073
• 35–39	0.0572	0.0335	0.088	0.0566	0.0336	0.092	0.0453	0.0335	0.176
• 40–49	0.0280	0.0477	0.558	0.0271	0.0477	0.570	0.0202	0.0489	0.680
Birth order (ref = first)									
• 2^nd^ to 4th	-0.0476	0.0155	0.002	-0.0475	0.0154	0.002	-0.0439	0.0137	0.001
• 5th or higher	-0.0667	0.0307	0.030	-0.0665	0.0306	0.030	-0.0620	0.0305	0.042
Worked (ref = Not worked)	0.0626	0.0481	0.193	0.0621	0.0481	0.197	0.0628	0.0518	0.226

*Primary analysis: Introduction of the policy estimated at 1 January 2007.

**Sensitivity analysis 1: Introduction of the policy estimated at 1 April 2007.

***Sensitivity analysis 2: Births between 1 January and 31 March 2007 deleted (Subsidy introduction, 1 January 2007)

The immediate effect of the subsidy varied depending on the rural household clusters. The lower the institutional delivery rate before the subsidy, the more the household clusters benefited from the policy (correlation of (-1) between the random effects of the intercept and subsidy). Thus, the delivery rate in health facilities increased by 44% (RR: 1.44, 95% CI 1.33–1.55) immediately after the introduction of the subsidy in the cluster with the lowest rate, compared with only 5% (RR: 1.05, 95% CI 0.98–1.14) for the cluster with the highest rate in the primary analysis.

Tables 4 and 5 present the relative (referred to as relative risk) and absolute changes (referred to as the rate differences) in the delivery rates in health facilities every 6 months after the introduction of the subsidy during the 42-month observation period. These results showed that changes are persistent and occurred faster in the rural areas than those in the urban areas. In the primary analysis, the delivery rate at health facilities increased by 12% (RR: 1.12, 95% CI 1.04–1.20) immediately after the introduction of the subsidy and then by 26% (RR: 1.26; 95% CI 0.86–1.86) after 42 months in the rural areas compared with 4% (RR: 1.04, 95% CI 0.98–1.10) and 13% (RR: 1.13, 95% CI 0.88–1.46), respectively, in the urban areas.

**Table 4:** Rate ratios (RRs) with corresponding 95% Confidence intervals (CIs) for associations between subsidy introduction and birth attendance in a health facility over time across urban and rural locations in Burkina Faso.

Time since subsidy introduction (months)	Primary analysis (Subsidy introduction: 1 January 2007)		Sensitivity analysis 1 (Subsidy introduction: 1 April 2007)		Sensitivity analysis 2 (Births between 1 January and 31 March 2007 deleted)	
	Urban	Rural	Urban	Rural	Urban	Rural
0	1.04 (0.98–1.10)	1.12 (1.04–1.20)	1.04 (1.00–1.08)	1.08 (1.02–1.14)	1.05 (1.00–1.10)	1.15 (1.06–1.24)
6	1.05 (0.97–1.14)	1.14 (1.01–1.27)	1.05 (1.00–1.10)	1.06 (0.97–1.16)	1.06 (0.99–1.14)	1.17 (1.04–1.31)
12	1.06 (0.96–1.18)	1.16 (0.99–1.36)	1.05 (0.98–1.13)	1.05 (0.93–1.18)	1.07 (0.97–1.19)	1.19 (1.01–1.39)
18	1.08 (0.94–1.23)	1.18 (0.96–1.44)	1.06 (0.97–1.16)	1.04 (0.89–1.20)	1.09 (0.95–1.24)	1.21 (0.99–1.48)
24	1.09 (0.93–1.28)	1.20 (0.93–1.54)	1.07 (0.95–1.20)	1.02 (0.85–1.23)	1.10 (0.93–1.29)	1.23 (0.96–1.58)
30	1.10 (0.91–1.34)	1.22 (0.91–1.64)	1.07 (0.94–1.23)	1.01 (0.81–1.25)	1.11 (0.91–1.35)	1.25 (0.93–1.69)
36	1.12 (0.89–1.40)	1.24 (0.88–1.75)	1.08 (0.92–1.27)	1.00 (0.78–1.28)	1.12 (0.89–1.41)	1.28 (0.91–1.80)
42	1.13 (0.88–1.46)	1.26 (0.86–1.86)	1.09 (0.91–1.30)	0.98 (0.74–1.31)	1.13 (0.87–1.47)	1.30 (0.88–1.92)

**Table 5:** Rate differences (RDs) with corresponding 95% Confidence intervals (CIs) for sssociations between subsidy introduction and birth attendance in a health facility over time across urban and rural locations in Burkina Faso.

Time since subsidy introduction (months)	Primary analysis (Subsidy introduction: 1 January 2007)		Sensitivity analysis 1 (Subsidy introduction: 1 April 2007)		Sensitivity analysis 2 (Births between 1 January and 31 March 2007 deleted)	
	Urban	Rural	Urban	Rural	Urban	Rural
0	3.4%(-1.4 to 8.2)	5.8%(2.4 to 9.2)	3.7% (0.6 to 6.8)	4.0% (1.2 to 6.7)	4.4% (0.4 to 8.5)	7.3%(4.1 to 10.5)
6	4.5%(-2.3 to 11.2)	6.9%(1.4 to 12.4)	4.2% (-1.0 to 8.6)	3.4% (-1.2 to 8.0)	5.4% (-0.8 to 11.6)	8.4%(3.1 to 13.8)
12	5.6%(-3.4 to 14.5)	8.1%(0.2 to 15.9)	4.8% (-1.3 to 10.9)	2.8% (-3.9 to 9.5)	6.4% (-2.3 to 15.0)	9.7%(1.9 to 17.4)
18	6.6%(-4.7 to 18.0)	9.2%(-1.1 to 19.7)	5.4% (-2.6 to 13.3)	2.1% (-7.0 to 11.3)	7.3% (-4.0 to 18.6)	10.9%(0.7 to 21.2)
24	7.7%(-6.1 to 21.6)	10.6%(-2.5 to 23.7)	5.9% (-4.0 to 15.9)	1.4% (-10.3 to 13.2)	8.3% (-5.7 to 22.3)	12.3%(-0.6 to 25.2)
30	8.8%(-7.5 to 25.2)	12.0%(-3.9 to 27.9)	6.5% (-5.5 to 18.5)	0.6% (-10.3 to 13.2)	9.3% (-7.4 to 26.0)	13.8%(-1.9 to 29.4)
36	10%(-9.0 to 28.9)	13.5%(-5.4 to 32.3)	7.1% (-7.0 to 21.1)	-0.2% (-18.0 to 17.6)	10.3% (-9.2 to 29.7)	15.3%(-3.3 to 33.9)
42	11.1%(-10.4 to 32.6)	15.0%(-6.9 to 37.0)	7.6% (-8.5 to 23.8)	-1.1% (-22.4 to 20.2)	11.3% (-10.9 to 33.5)	16.9%(-4.7 to 38.5)

Neonatal mortality did not significantly decrease immediately after the introduction of the policy or in the long term (for the coefficients, see S2 Table). Table 6 shows the relative and absolute changes in neonatal mortality rates every 6 months after implementation of the subsidy. Under the considered scenario, mortality decreased immediately after the introduction of the subsidy between 2 and 4 neonatal deaths per 1,000 live births (i.e., a 7 to 13% relative decrease). These differences were not significant. Mortality decreased by 9 deaths per 1,000 live births at 42 months after subsidy implementation, which was a non-significant decrease of 19%.

**Table 6:** Rate ratios (RRs) and Rate differences (RDs) with corresponding 95% Confidence intervals (CIs) for associations between subsidy introduction and neonatal mortality over time in Burkina Faso.

Time since subsidy introduction (months)	Primary analysis (Subsidy introduction: 1 January 2007)		Sensitivity analysis 1 (Subsidy introduction: 1 April 2007)		Sensitivity analysis 2 (Births between 1 January and 31 March 2007 deleted)	
	RR (95% CI)	RD (95% CI)	RR (95% CI)	RD (95% CI)	RR (95% CI)	RD ((95% CI)
0	0.93 (0.61–1.44)	-0.2%(-1.6 to 1.2%)	0.88 (0.56–1.38)	-0.4%(-1.8 to 1.0%)	0.87 (0.55–1.39)	-0.4%(-1.9 to 1.0%)
6	0.90 (0.61–1.33)	-0.3%(-1.6 to 0.9%)	0.85 (0.57–1.27)	-0.5%(-1.7 to 0.7%)	0.85 (0.56–1.28)	-0.5%(-1.8 to 0.8%)
12	0.86 (0.60–1.24)	-0.4%(-1.6 to 0.7%)	0.83 (0.58–1.18)	-0.6%(-1.7 to 0.5%)	0.82 (0.57–1.19)	-0.6%(-1.7 to 0.6%)
18	0.83 (0.60–1.16)	-0.5%(-1.6 to 0.5%)	0.80 (0.58–1.10)	-0.6%(-1.6 to 0.4%)	0.80 (0.57–1.12)	-0.6%(-1.7 to 0.4%)
24	0.80 (0.58–1.11)	-0.6%(-1.7 to 0.4%)	0.78 (0.57–1.05)	-0.7%(-1.7 to 0.3%)	0.78 (0.57–1.07)	-0.7%(-1.7 to 0.3%)
30	0.77 (0.56–1.07)	-0.7%(-1.8 to 0.3%)	0.76 (0.56–1.03)	-0.8%(-1.8 to 0.2%)	0.75 (0.55–1.04)	-0.8%(-1.8 to 0.3%)
36	0.74 (0.53–1.04)	-0.8%(-1.9 to 0.3%)	0.73 (0.53–1.02)	-0.8%(-1.9 to 0.2%)	0.73 (0.53–1.02)	-0.8%(-1.9 to 0.2%)
42	0.71 (0.49–1.03)	-0.9%(-2.0 to 0.3%)	0.71 (0.49–1.03)	-0.9%(-2.0 to 0.2%)	0.71 (0.49–1.03)	-0.9%(-2.0 to 0.2%)

## Discussion

Despite numerous publications on the subject [5–8, 10, 12, 17, 19], the effects of public policies to reduce financial barriers to health care, especially at the national level, still must be thoroughly assessed in most sub-Saharan countries. To the best of our knowledge, this report is the fourth study to attempt to assess the impact of these policies on neonatal mortality. However, this study is the first to consider potential effect variation based on household socioeconomic characteristics and secular trends through geographical units, as recommended by French et al [32].

Our findings suggested that the delivery subsidy in Burkina Faso was associated with an increase in institutional deliveries immediately after the introduction of the subsidy between 4% and 5% in urban areas and between 8% and 15% in rural areas depending on the envisaged scenario for the policy introduction date. The decrease in neonatal mortality after the implementation of this policy remained limited and non-significant in this study.

These findings confirm the results of McKinnon et al. [10]. Their study also found that these policies were associated with a non-significant decrease of 2.9 deaths per 1,000 live births (95% CI -6.8; 1.0) and a significant 5% increase in health facility deliveries in three countries (Ghana, Senegal and Kenya). In contrast, the two other studies support that these policies were associated with significant reductions in neonatal mortality in Nepal [18], Mali and Benin [19].

An increase in facility-based delivery only is not sufficient to reduce maternal and neonatal mortality. A high level of quality care is needed to treat appropriately obstetric and neonatal complications [4, 40]. In that respect, in Mali and Benin, where a reduction in neonatal mortality was reported (adjusted OR = 0.70; 95% CI 0.58–0.85) to be associated with the cost-reduction policies, an increase in caesarean section (C-section) rates (adjusted OR = 1.36; 95% CI = 1.11–1.66) has also been reported [19]. Conversely, in Ghana, Senegal and Kenya, where McKinnon et al. [10] found no significant association between neonatal mortality reduction and the introduction of cost-reduction policies, the authors found no increase in C-section rates associated with these policies. In Burkina Faso, subsidy is reported to be associated with a slight increase in C-section rates by 0.70% [8]. We have no information on the trends in quality of care in primary health care facilities and referral hospitals during the study period. However, we assume that the lack of marked improvement in quality of care could explain the lack of effect of the cost-reduction policy on neonatal mortality.

Two previous studies [6, 8] in Burkina Faso used DHS consecutive data in an aggregated form and reported a significant increase of 25% after the subsidy in the first study and a 4% slope change per year in the second study. These studies used a long pre-intervention period (1988 or 1990 to 2007) but did not discuss whether the secular trend experienced significant variations [41]. Indeed, the results of these two studies suggested that changes in the secular trend slope occurred between 1988 and 2007. Ridde et al. [12] also reported an increase in health facility deliveries three years before the introduction of the subsidy.

A recent assessment [7] conducted in two semi-urban districts demonstrated greater and persistent effects 24 months later. Various studies have mentioned heterogeneity of subsidy effects between health districts [12] and health centres within the same district [21]. Our study asserts that this heterogeneity occurs at an even more geographically restricted level (the cluster = enumeration area) in rural areas, where the enumeration area usually represents a village.

According to our findings, the subsidy would also be associated with a reduction in health access inequalities between urban and rural areas and among rural villages.

We found no interaction between the subsidy and household wealth. All socioeconomic strata benefited from the effects of the subsidy, as shown in a rural district of Burkina [17]. McKinnon et al. [9] also found no interaction in three sub-Saharan African countries using DHSs and the same categorization of household wealth. However, other studies [5, 7, 8] argued that this cost-cutting was more beneficial for poor people. In the case of these studies, the household wealth was locally or individually built in rural and urban areas. This situation may be more appropriate because it considers the local context, as opposed to our study, which classifies the household comparatively to the other households of the overall country and can result in overly heterogeneous categories. In addition, the use of fixed percentiles in our study to create wealth categories from a continuous variable can lead to similar groups in the case of a high concentration in a narrow range and then provide misleading results [42].

There are several limitations to the interpretation of our results. First, the data were derived from a cross-sectional survey conducted in 2010. However, the subsidy also aimed to reduce maternal mortality. If the intervention actually was associated with a reduction in maternal mortality, our results would underestimate the reduction in neonatal mortality associated with the subsidy. In fact, maternal death could increase by up to nine times the risk of neonatal death [43]. As a result, these deaths would be underreported in the pre-intervention compared to the post-intervention period. The last three DHSs showed a continuous decline in the maternal mortality rate, with an apparently unchanged trend after the introduction of the subsidy. Therefore, we believe that our results are not altered by a significant decrease in maternal mortality.

Second, outcome variables are self-reported and may be subject to potential misclassification, especially for neonatal deaths, which can be mistaken for a stillborn delivery when they occur within the first 24 hours [43]. However, the sensitivity analyses show that the results are sufficiently robust in the presence of misclassification of exposure.

Third, some potential confounders of neonatal death as breastfeeding, birth weight and quality of care were not included in the analyses.

Furthermore, Rutstein et al. [26] considered that the household assets used to assess household wealth and education generally experienced few variations over short periods and that limited variable misclassifications could be obtained with a limited 10-year observation period for neonatal deaths and a 5-year period for delivery in health facilities.

Our inference is on population neonatal mortality; if we limited our analysis to women who gave birth in health centres, this inference could be false if many neonatal deaths occurred in persistent home deliveries. According to Rothman et al. [42], the validity of the ecological effect in this case depends on the capacity to control the differences between the before and after groups. By using individual data and an individual level analysis, we were able to incorporate as many confounding variables as possible to reduce the risk of bias.

Finally, to the best of our knowledge, no major intervention for maternal and neonatal health was implemented around the subsidy introduction date. Therefore, immediate effects can reasonably be attributed to the subsidy policy. However, local initiatives to extend the national subsidy and ensure free delivery care have been completed by this mechanism in several districts [22, 23]. Therefore, the effects observed after the first months can no longer be attributed solely to the subsidy in certain areas.

## Conclusion

Our study suggests that the national subsidy for deliveries and emergency obstetric and neonatal care in Burkina Faso is associated with short-term increases in deliveries in health facilities, particularly in rural areas, and with a non-significant decrease in neonatal mortality. This policy has benefited the rich as well as the poor, and these effects are independent of the women’s education levels. Since the subsidy has evolved in 2016 towards a delivery expenses total exemption, further studies are required to assess the long-term impact of the Reproductive Health financing policy in Burkina Faso.

## Supporting information

S1 Table Sociodemographic and economic characteristics of live births between June 2005 and December 2010 in Burkina Faso. (DOCX)

S2 Table Estimates for the effects of the national subsidy on neonatal mortality in Burkina Faso. (DOCX)

## References

[1] Gabrysch S, Campbell OM. Still too far to walk: literature review of the determinants of delivery service use. BMC Pregnancy Childbirth. 2009;9:34 10.1186/1471-2393-9-34 . 19671156 PMC2744662

[2] Campbell OM, Graham WJ. Strategies for reducing maternal mortality: getting on with what works. Lancet. 2006;368(9543):1284–99. 10.1016/S0140-6736(06)69381-1 . 17027735

[3] UNICEF. monitoring the situation of children and women: delivery care. New York: United Nations Children’s Fund, 2015.

[4] Richard F, Witter S, de Brouwere V. Innovative approaches to reducing financial barriers to obstetric care in low-income countries. Am J Public Health. 2010;100(10):1845–52. 10.2105/AJPH.2009.179689 . 20724689 PMC2936984

[5] Dzakpasu S, Soremekun S, Manu A, Ten Asbroek G, Tawiah C, Hurt L, et al Impact of free delivery care on health facility delivery and insurance coverage in Ghana’s Brong Ahafo Region. PLoS One. 2012;7(11):e49430 10.1371/journal.pone.0049430 . 23173061 PMC3500286

[6] Ganaba R, Ilboudo PGC, Cresswell JA, Yaogo M, Diallo CO, Richard F, et al The obstetric care subsidy policy in Burkina Faso: what are the effects after five years of implementation? Findings of a complex evaluation. BMC Pregnancy Childbirth. 2016;16(1):84 10.1186/s12884-016-0875-2 27101897 PMC4840487

[7] Langlois EV, Karp I, Serme JD, Bicaba A. Effect of a policy to reduce user fees on the rate of skilled birth attendance across socioeconomic strata in Burkina Faso. Health Policy Plan. 2015;31(4):462–71. 10.1093/heapol/czv088 . 26453087 PMC4986241

[8] Leone T, Cetorelli V, Neal S, Matthews Z. Financial accessibility and user fee reforms for maternal healthcare in five sub-Saharan countries: a quasi-experimental analysis. BMJ Open. 2016;6(1):e009692 10.1136/bmjopen-2015-009692 . 26823178 PMC4735164

[9] McKinnon B, Harper S, Kaufman JS. Who benefits from removing user fees for facility-based delivery services? Evidence on socioeconomic differences from Ghana, Senegal and Sierra Leone. Soc Sci Med. 2015;135:117–23. 10.1016/j.socscimed.2015.05.003 25965892

[10] McKinnon B, Harper S, Kaufman JS, Bergevin Y. Removing user fees for facility-based delivery services: a difference-in-differences evaluation from ten sub-Saharan African countries. Health policy and planning. 2014;30(4):431–42. 10.1093/heapol/czu027 24816570 PMC4385820

[11] Philibert A, Ravit M, Ridde V, Dossa I, Bonnet E, Bedecarrats F, et al Maternal and neonatal health impact of obstetrical risk insurance scheme in Mauritania: a quasi experimental before-and-after study. Health policy and planning. 2017;32(3):405–17. 10.1093/heapol/czw142 . 27935801 PMC5886239

[12] Ridde V, Richard F, Bicaba A, Queuille L, Conombo G. The national subsidy for deliveries and emergency obstetric care in Burkina Faso. Health policy and planning. 2011;26 Suppl 2:ii30–40. 10.1093/heapol/czr060 . 22027917

[13] Witter S, Boukhalfa C, Cresswell JA, Daou Z, Filippi V, Ganaba R, et al Cost and impact of policies to remove and reduce fees for obstetric care in Benin, Burkina Faso, Mali and Morocco. Int J Equity Health. 2016;15(1):123 10.1186/s12939-016-0412-y . 27483993 PMC4970227

[14] Dzakpasu S, Powell-Jackson T, Campbell OMR. Impact of user fees on maternal health service utilization and related health outcomes: a systematic review. Health Policy Plan. 2014;29(2):137–50. 10.1093/heapol/czs142 23372035

[15] HattL, MakinenM, MadhavanS, ConlonC. Effects of user fee exemptions on the provision and use of maternal health services: a review of literature. J Health Popul Nutr. 2013;31(4 (suppl 2)):S67–S80.24992804

[16] Lagarde M, Palmer N. The impact of user fees on access to health services in low- and middle-income countries. The Cochrane database of systematic reviews. 2011;(4):Cd009094 10.1002/14651858.CD009094 . 21491414 PMC10025428

[17] De Allegri M, Ridde V, Louis VR, Sarker M, Tiendrebeogo J, Ye M, et al The impact of targeted subsidies for facility-based delivery on access to care and equity—Evidence from a population-based study in rural Burkina Faso. J Public Health Policy. 2012;33(4):439–53. 10.1057/jphp.2012.27 . 22932023

[18] Lamichhane P, Sharma A, Mahal A. Impact evaluation of free delivery care on maternal health service utilisation and neonatal health in Nepal. Health Policy Plan. 2017;32(10):1427–36. 10.1093/heapol/czx124 . 29029159

[19] Ravit M, Audibert M, Ridde V, de Loenzien M, Schantz C, Dumont A. Removing user fees to improve access to caesarean delivery: a quasi-experimental evaluation in western Africa. BMJ Glob Health. 2018;3(1):e000558 10.1136/bmjgh-2017-000558 . 29515916 PMC5838396

[20] Institut National de la Statistique et de la Démographie (INSD), ICF International. The Burkina Faso Demographic and Health and Multiple Cluster Indicator Survey 2010. Calverton, Maryland, USA: INSD et ICF International, 2012.

[21] Belaid L, Ridde V. Contextual factors as a key to understanding the heterogeneity of effects of a maternal health policy in Burkina Faso? Health policy and planning. 2015;30(3):309–21. 10.1093/heapol/czu012 . 24633914 PMC4353895

[22] Kouanda S, Bado A, Meda IB, Yameogo GS, Coulibaly A, Haddad S. Home births in the context of free health care: the case of Kaya health district in Burkina Faso. Int J Gynaecol Obstet. 2016;135 Suppl 1:S39–S44. 10.1016/j.ijgo.2016.08.009 . 27836083

[23] Meda IB, Zoungrana Y, Bado A, Kouanda S. Émergence de politiques locales d’exemption dans un contexte national de subvention dans le district sanitaire de Kaya, Burkina Faso. In: Santé maternelle et accès aux soins en Afrique de l’Ouest: L’Harmattan; 2013. p. 163–86.

[24] Ridde V, Kouanda S, Yameogo M, Kadio K, Bado A. Why do women pay more than they should? A mixed methods study of the implementation gap in a policy to subsidize the costs of deliveries in Burkina Faso. Eval Program Plann. 2013;36(1):145–52. 10.1016/j.evalprogplan.2012.09.005 . 23123308

[25] Lawn JE, Kerber K, Enweronu-Laryea C, Cousens S. 3.6 million neonatal deaths—what is progressing and what is not? Semin Perinatol. 2010;34(6):371–86. 10.1053/j.semperi.2010.09.011 . 21094412

[26] Rutstein SO, Johnson K. DHS Comparative reports No.6: The DHS wealth index: Calverton, MD: ORC Macro; 2004.

[27] Mosley WH, Chen LC. An analytical framework for the study of child survival in developing countries. 1984. Bull World Health Organ. 2003;81(2):140–5. . 12756980 PMC2572391

[28] Yelland LN, Salter AB, Ryan P. Performance of the modified poisson regression approach for estimating relative risks from clustered prospective data. Am J Epidemiol. 2011;174(8):984–92. 10.1093/aje/kwr183 21841157

[29] Zou G. A modified poisson regression approach to prospective studies with binary data. Am J Epidemiol. 2004;159(7):702–6. 10.1093/aje/kwh090 15033648

[30] Lagarde M. How to do (or not to do) … Assessing the impact of a policy change with routine longitudinal data. Health policy and planning. 2012;27(1):76–83. 10.1093/heapol/czr004 21278077

[31] Wagner AK, Soumerai SB, Zhang F, Ross-Degnan D. Segmented regression analysis of interrupted time series studies in medication use research. J Clin Pharm Ther. 2002;27(4):299–309. 10.1046/j.1365-2710.2002.00430.x 12174032

[32] French B, Heagerty PJ. Analysis of longitudinal data to evaluate a policy change. Statistics in medicine. 2008;27(24):5005–25. Epub 2008/07/12. 10.1002/sim.3340 . 18618416 PMC3415557

[33] Nguyen HT, Zombré D, Ridde V, De Allegri M. The impact of reducing and eliminating user fees on facility-based delivery: a controlled interrupted time series in Burkina Faso. Health Policy and Planning. 2018;33(8):948–56. 10.1093/heapol/czy077 30256941

[34] Serumaga B, Ross-Degnan D, Avery AJ, Elliott RA, Majumdar SR, Zhang F, et al Effect of pay for performance on the management and outcomes of hypertension in the United Kingdom: interrupted time series study. BMJ. 2011;342:d108 10.1136/bmj.d108 21266440 PMC3026849

[35] Taljaard M, McKenzie JE, Ramsay CR, Grimshaw JM. The use of segmented regression in analysing interrupted time series studies: an example in pre-hospital ambulance care. Implement Sci. 2014;9(1):77 10.1186/1748-5908-9-77 . 24943919 PMC4068621

[36] Rabe-Hesketh S, Skrondal A. Multilevel and longitudinal modeling using stata, Volumes I and II 3rd ed College Station, TX: Taylor & Francis; 2012.

[37] Ahern J, Hubbard A, Galea S. Estimating the effects of potential public health interventions on population disease burden: a step-by-step illustration of causal inference methods. Am J Epidemiol. 2009;169(9):1140–7. 10.1093/aje/kwp015 19270051 PMC2732980

[38] Greenland S. Model-based estimation of relative risks and other epidemiologic measures in studies of common outcomes and in case-control studies. Am J Epidemiol. 2004;160(4):301–5. 10.1093/aje/kwh221 . 15286014

[39] Cummings P. Methods for estimating adjusted risk ratios. Stata J. 2009;9(2):175–96.

[40] StantonME, HiggsES, KoblinskyM. Investigating financial incentives for maternal health: an introduction. J Health Popul Nutr. 2013;31(4 Suppl 2):1–7. .24992799

[41] Lopez Bernal J, Cummins S, Gasparrini A. Interrupted time series regression for the evaluation of public health interventions: a tutorial. International journal of epidemiology. 2016;46(1):348–55. 10.1093/ije/dyw098 . 27283160 PMC5407170

[42] Rothman KJ, Greenland S, Lash T. Modern epidemiology. 3rd ed Philadelphia: Lippincott Williams & Wilkins; 2008.

[43] Ronsmans C, Chowdhury ME, Dasgupta SK, Ahmed A, Koblinsky M. Effect of parent’s death on child survival in rural Bangladesh: a cohort study. Lancet. 2010;375(9730):2024–31. 10.1016/S0140-6736(10)60704-0 . 20569842

