# Supplementary material for: Impact of fee subsidy policy on perinatal health in a low-resource setting: A quasi-experimental study
Source: PLoS One. 2018 Nov 8;13(11):e0206978. doi: 10.1371/journal.pone.0206978 (PMC6224097; doi:10.1371/journal.pone.0206978)
Supplement: S1 Table — (DOCX) [file pone.0206978.s001.docx]

S1 Table*: Sociodemographic and economic characteristics of live births by area of residence between June 2005 and December 2010 in Burkina Faso (Demographic and Health Survey 2010).

|  | Urban | | Rural | |
| --- | --- | --- | --- | --- |
| Characteristic | Pre-subsidy | Post-subsidy | Pre-subsidy | Post-subsidy |
|  | n = 761 | n= 2,418 | n = 2,635 | n = 8,939 |
| Place of birth (health facility) | 659 (86.6) | 2,243 (92.8) | 1,327 (50.4) | 5,835 (65.3) |
| Number of births (multiple) | 12 (1.6) | 51 (2.1) | 46 (1.8) | 178 (2.0) |
| Woman’s age (years |  |  |  |  |
| - 15-19 | 96 (12.6) | 295 (12.2) | 350 (13.3) | 1,025 (11.5) |
| - 20-24 | 238 (31.3) | 702 (29.0) | 677 (25.7) | 2,355 (26.3) |
| - 25-29 | 197 (25.9) | 664 (27.5) | 666 (25.3) | 2,280 (25.5) |
| - 30-34 | 126 (16.5) | 434 (17.9) | 476 (18.1) | 1,607 (18.0) |
| - 35-39 | 76 (10.0) | 213 (8.8) | 325 (12.3) | 1,113 (12.5) |
| - 40-49 | 28 (3.7) | 110 (4.6) | 141 (5.3) | 559 (6.2) |
| Birth order |  |  |  |  |
| - First | 220 (28.9) | 642 (26.5) | 450 (17.1) | 1,500 (16.8) |
| - 2^nd^ to 4th | 368 (48.4) | 1,274 (52.7) | 1,193 (45.3) | 4,027 (45.0) |
| - 5th or higher | 173 (22.7) | 502 (20.8) | 992 (37.6) | 3,412 (38.2) |
| Woman’s literacy (illiterate) | 487 (64.0) | 1,536 (63.5) | 2,464 (93.5) | 8,233 (92.1) |
| Woman’s education |  |  |  |  |
| - None | 424 (55.7) | 1,383 (57.2) | 2,436 (92.5) | 8,062 (90.2) |
| - Primary | 198 (26.0) | 583 (24.1) | 164 (6.2) | 708 (7.9) |
| - Secondary or higher | 139 (18.3) | 452 (18.7) | 35 (1.3) | 169 (1.9) |
| Woman’s occupation (working) | 560 (73.6) | 1,704 (70.5) | 2,176 (82.6) | 7,294 (81.6) |
| Household wealth |  |  |  |  |
| - Poorest | 31 (4.1) | 93 (3.8) | 653 (24.8) | 2,115 (23.7) |
| - Poorer | 36 (4.7) | 127 (5.2) | 673 (25.5) | 2,272 (25.4) |
| - Middle | 60 (7.9) | 220 (9.1) | 649 (24.6) | 2,311 (25.9) |
| - Richer | 184 (24.2) | 579 (24.0) | 550 (20.9) | 1,862 (20.8) |
| - Richest | 450 (59.1) | 1399 (57.9) | 110 (4.2) | 379 (4.2) |

*****Subsidy start date: 1 January 2007

****** The 40-44 and 45-49 age groups were merged due to their low numbers.
