# Supplementary material for: Impact of fee subsidy policy on perinatal health in a low-resource setting: A quasi-experimental study
Source: PLoS One. 2018 Nov 8;13(11):e0206978. doi: 10.1371/journal.pone.0206978 (PMC6224097; doi:10.1371/journal.pone.0206978)
Supplement: S2 Table — (DOCX) [file pone.0206978.s002.docx]

Table S2: Estimates for different two-level random-intercept (RI) Poisson regression models for the effects of the national subsidy on neonatal mortality in Burkina Faso (Demographic and Health Survey 2010).

| Characteristics | Primary analysis* | | Sensitivity analysis 1** | | Sensitivity analysis 2*** | |
| --- | --- | --- | --- | --- | --- | --- |
|  | Estimate (SE) | p-value | Estimate (SE) | p-value | Estimate (SE) | p-value |
| Non-adjusted coefficient | | | | | | |
| Subsidy | -0.3916 (0.0643) | <0.001 | -0.4174 (0.0721) | <0.001 | -0.4193 (0.0715) | <0.001 |
| Fully adjusted coefficients | | | | | | |
| Intercept | -3.8531 (0.1512) | <0.001 | -3.8538 (0.1453) | <0.001 | -3.8128 (0.1532) | <0.001 |
| Subsidy | -0.0681 (0.2204) | 0.757 | -0.1298 (0.2310) | 0.574 | -0.1359 (0.2383) | 0.568 |
| Time | -0.0023 (0.0021) | 0.276 | -0.0022 (0.0019) | 0.244 | -0.0022 (0.0021) | 0.275 |
| Post-time | -0.0064 (0.0056) | 0.255 | -0.0050 (0.0066) | 0.447 | -0.0049 (0.0065) | 0.452 |
| Illiterate woman (ref: Literate) | 0.1711 (0.1002) | 0.088 | 0.1716 (0.1008) | 0.089 | 0.1657 (0.1073) | 0.123 |
| Household wealth (ref = Poorest) |  |  |  |  |  |  |
| - Poorer | 0.2589 (0.0757) | 0.001 | 0.2589 (0.0758) | 0.001 | 0.2521 (0.0801) | 0.002 |
| - Middle | 0.1514 (0.1076) | 0.159 | 0.1521 (0.1070) | 0.155 | 0.1366 (0.1122) | 0.224 |
| - Richer | 0.0547 (0.0952) | 0.566 | 0.0545 (0.0950) | 0.566 | 0.0440 (0.0964) | 0.648 |
| - Richest | -0.1694 (0.1299) | 0.192 | -0.1691 (0.1298) | 0.193 | -0.1740 (0.1321) | 0.188 |
| Multiple births (ref = Single) | 1.8700 (0.1026) | <0.001 | 1.8669 (0.1107) | <0.001 | 1.8794 (0.1028) | <0.001 |
| Newborn male (ref: Female) | 0.1863 (0.0711) | 0.009 | 0.1866 (0.0710) | 0.009 | 0.1996 (0.0665) | 0.003 |
| Woman’s age (ref = 15-19) |  |  |  |  |  |  |
| - 20-24 | -0.4870 (0.0942) | <0.001 | -0.4875 (0.0941) | <0.001 | -0.5184 (0.0903) | <0.001 |
| - 25-29 | -0.4004 (0.1060) | <0.001 | -0.4006 (0.1060) | <0.001 | -0.4340 (0.1082) | <0.001 |
| - 30-34 | -0.3646 (0.1133) | 0.001 | -0.3659 (0.1138) | 0.001 | -0.3946 (0.1106) | 0.001 |
| - 35-39 | -0.2567 (0.1150) | 0.026 | -0.2584 (0.1150) | 0.025 | -0.3070 (0.1116) | 0.006 |
| - 40-49 | 0.1581 (0.2054) | 0.442 | 0.1577 (0.2054) | 0.443 | 0.1194 (0.2158) | 0.580 |
| Previous birth interval (ref: >= 36 months) |  |  |  |  |  |  |
| - < 36 months | 0.6887 (0.0842) | <0.001 | 0.6881 (0.0835) | <0.001 | 0.6823 (0.0791) | <0.001 |
| - 1st birth | 0.8160 (0.1155) | <0.001 | 0.8155 (0.1152) | <0.001 | 0.7918 (0.1114) | <0.001 |
| Working woman (ref = Not working) | -0.0384 (0.0644) | 0.551 | -0.0388 (0.0646) | 0.548 | -0.0250 (0.0640) | 0.697 |
| Rural residence of household (ref = Urban) | -0.0997 (0.1002) | 0.320 | -0.1001 (0.1002) | 0.318 | -0.1046 (0.0997) | 0.294 |
| Random parts | | | | | | |
| $\sqrt{\psi_{11}}$ | 0.3734 | | 0.3738 | | 0.3558 | |

*Primary analysis: 1 January 2007 (Subsidy introduction).

**Sensitivity analysis 1: 1 April 2007 (Subsidy introduction).

***Sensitivity analysis 2: Births between 1 January and 31 March 2007 deleted (Subsidy introduction 1 April 2007).
